# Cultural differences in the production of emotional facial expressions: a review

**DOI:** 10.3389/fpsyg.2026.1699374

**Published:** 2026-02-02

**Authors:** Adèle Gallant, Marie-Pier Mazerolle, Annalie Pelot, Annie Roy-Charland

**Affiliations:** 1 School of Psychology, Université de Moncton, Moncton, NB, Canada; 2 Department of Psychology, Laurentian University, Sudbury, ON, Canada

**Keywords:** cultural differences, culture, emotional facial expressions, encoding, production

## Abstract

Whereas cross-cultural differences in recognition of emotional facial expressions are widely established, less information exists regarding differences in their production. The current systematic review was conducted to summarize and clarify the role of culture in the production of emotional facial expressions. Following a two-step process, 21 peer-reviewed articles were included within the current review, from which four categories were generated exploring findings from child/infant and adult studies separately: (1) production of spontaneous expressions, (2) production of posed expressions, (3) comparison of spontaneous and posed expressions, and (4) others. Whereas the production of emotional facial expressions is shown to be largely universal, culturally variable nuances exist but vary according to spontaneous versus posed expressions.

## 1 Introduction

Emotional facial expressions are among the most salient channels of non-verbal communication. By activating specific muscle groups, humans convey internal states, guide social interactions, and provide environmental cues to others (Darwin, 1998; Ekman, 1971; Horstmann, 2003; Sander and Scherer, 2009). A smile can signal happiness or approval (Brown and Moore, 2002; Rychlowska et al., 2017), while widened eyes and a gaping mouth may communicate fear, alerting others to possible danger (Gosselin and Kirouac, 1995; Lee et al., 2013). Because of their communicative power, emotional facial expressions have long been central to research on human emotion.

### 1.1 Historical and theoretical background

Charles Darwin argued that emotional facial expressions are innate, biologically grounded, and evolutionarily conserved across species and human groups (Darwin, 1998). His proposal that emotions are adaptive responses with characteristic behavioral displays inspired discrete emotion theories, which posit that a limited set of basic emotions are universally shared, each linked to distinct eliciting conditions, physiological changes, and observable expressions (Izard, 1971; Tomkins and McCarter, 1964; Magai, 2001).

Building on this framework, Ekman et al. (2002) developed the Facial Action Coding System (FACS) to identify muscular movements (action units, AUs) underlying visible appearance changes in the face. This tool enabled researchers to chart reproducible patterns of muscle activation associated with discrete emotions. For instance, AU6 (cheek raiser) and AU12 (lip corner puller) were seen as cues happiness, while the combination of AU4 (brow lowerer) and AU7 (lid tightener) are associated with anger. Many researchers have since identified combinations of AUs that recur reliably with particular emotions (Ekman et al., 2002).

### 1.2 Universality of emotional facial expressions

With the theories such as the basic emotion theory (Ekman et al., 1969), the universality hypothesis argues that basic emotions are expressed and recognized similarly across cultures. Early theorists such as Tomkins and McCarter (1964) proposed innate affect programs that generate recognizable expressions. Ekman and Friesen (1971) famously asked members of a preliterate group in Papua New Guinea to produce expressions corresponding to emotional scenarios (e.g., “show me what you would look like if your child had died”), and found that American participants recognized them with high accuracy. Conversely, Papua New Guinea participants reliably recognized Americans’ posed expressions. These findings were widely interpreted as evidence that six emotions, anger, disgust, fear, sadness, surprise, and happiness, constitute “basic emotions” with universal facial signals (Ekman et al., 1969; Ekman, 1971, 1992; Ekman and Rosenberg, 1997; Izard, 1971, 1994; Hwang and Matsumoto, 2015). The universality perspective has influenced psychology, anthropology, and neuroscience, as well as clinical domains such as assessment (Douglas and Porter, 2010; Oosterman et al., 2016) and interventions (Webster et al., 2021).

### 1.3 Culture and emotional facial expressions

Despite its dominance, the universality hypothesis has been challenged by accounts emphasizing cultural variability. Cultural constructionist theories argue that emotional facial expressions are not biologically fixed “readouts” but are shaped by learned values, beliefs, norms, meanings, language, and customs (Triandis, 1995; Mascolo et al., 2003; Shaver et al., 1992; Russell, 1994, 1995). In this view, culture not only regulates expressions but defines the meaning of emotions themselves. Several mechanisms have been proposed. For instance, display rules are culturally learned guidelines that dictate how and when emotions should be shown, concealed, or exaggerated (Ekman and Friesen, 1975; Matsumoto, 1990; Banerjee, 1997; Banerjee and Yuill, 1999; Matsumoto and Hwang, 2013). For example, Asian cultural contexts place emphasis on shame and social harmony, encouraging individuals to mask negative emotions and exaggerate positive ones (Fung, 1999; Shaver et al., 1992; Camras et al., 1998, 2006; Mui et al., 2017). Another mechanism is the in-group advantage: people are more accurate at recognizing emotional facial expressions from members of their own cultural group (Elfenbein and Ambady, 2002; Elfenbein et al., 2004). A meta-analysis supported this effect across many emotions, and further research suggests culturally specific “non-verbal accents” in emotional facial expressions (Elfenbein et al., 2002). For example, smiles may be universal markers of happiness, but cultures differ in whether closed- versus open-mouth smiles are common (Beaupré and Hess, 2003; Bjornsdottir and Rule, 2021). Together, these findings highlight that cultural learning and socialization might shape both the production and recognition of emotional facial expressions.

### 1.4 Methodological sources of discrepancy

While the reason being the inconsistencies in the literature leading to the persistence of the debate is unclear, methodological heterogeneity might play an important role. Amongst methodological differences, task designs might matter. Forced-choice paradigms, which constrain participants to a fixed set of labels, tend to inflate recognition accuracy, whereas free-labeling tasks produce lower accuracy and seem to reveal more culturally variable interpretations (Russell, 1994, 1995; Nelson and Russell, 2013; Ortony, 2022). Furthermore, the operationalizations of “culture” further complicate conclusions. Studies often substitute nationality for culture, grouping heterogeneous populations into broad categories (e.g., “East Asian”), ignoring important intracultural variation (Hofstede, 2001). Research using international students raises similar concerns, as acculturation may rapidly alter recognition accuracy (Elfenbein and Ambady, 2003). Another important variation between studies is related to the emotional facial expressions themselves. Many influential studies have used posed expressions, deliberately enacted by actors or participants under instruction. These could lead to important difference in stimuli both between studies but also within studies. While researchers can control for the presence of specific muscle movements in their stimuli, not all do. Indeed, posed expressions are produced by explicit instructions to display a specific emotion or activate specific muscles, while spontaneous expressions result from an induced emotional experience. While standardizable, posed expressions are typically produced in controlled laboratory settings and may differ systematically from spontaneous expressions, which arise from genuine emotional experiences often captured in naturalistic or ecologically valid contexts (Namba et al., 2017). Expressions may also differ as a function of the study setting. In this sense, non-naturalistic studies can be defined as those conducted in controlled laboratory environments, whereas naturalistic studies involve the observation of emotional expressions in real-life contexts. It is possible that the setting influences the expressions produced. However, context does not necessarily determine the elicitation method: for example, a participant may produce a spontaneous expression in response to an emotional film clip, even though the situation takes place in a non-naturalistic laboratory setting. Recognition accuracy and cultural differences can vary depending on which type of stimulus is used (Matsumoto and Hwang, 2017). These methodological inconsistencies likely explain why some studies strongly support universality while others report cultural divergence (e.g., Blais et al., 2008; Haidt and Keltner, 1999; Jack et al., 2009, 2012; Elfenbein and Ambady, 2002).

### 1.5 The role of production procedures

Notably, most cross-cultural studies focus on recognition, while the production of expressions has received comparatively little attention. Yet production determines the very stimuli on which recognition studies rely. If expressions differ across cultures in intensity, timing, or AU configuration, whether due to innate mechanisms, display rules, or methodological elicitation, then recognition findings alone cannot resolve whether expressions are universal or culturally variable. For instance, a recognition study comparing Western and East Asian observers may find accuracy differences. Without knowing whether the expressions judged were posed or spontaneous, or how they were originally produced within each cultural context, it is impossible to determine whether these differences reflect perceptual biases, cultural display rules, or deeper biological variation. In this sense, production is not simply parallel to recognition, it is foundational to understanding the stimuli that have fueled this debate.

### 1.6 The goal of the review

The present systematic review focuses on cross-cultural production of emotional facial expressions. By synthesizing studies across development and adulthood, and comparing methodological approaches (posed vs. spontaneous, static vs. dynamic, task designs, cultural groupings), we aim to clarify whether cultural influences on production contribute to the inconsistencies that characterize the universality vs. cultural variation debate. By examining production directly, we seek to move beyond recognition alone, toward a more integrated understanding of how universal biological mechanisms and cultural processes jointly shape the human face of emotion.

## 2 Methods

**FIGURE 1:**
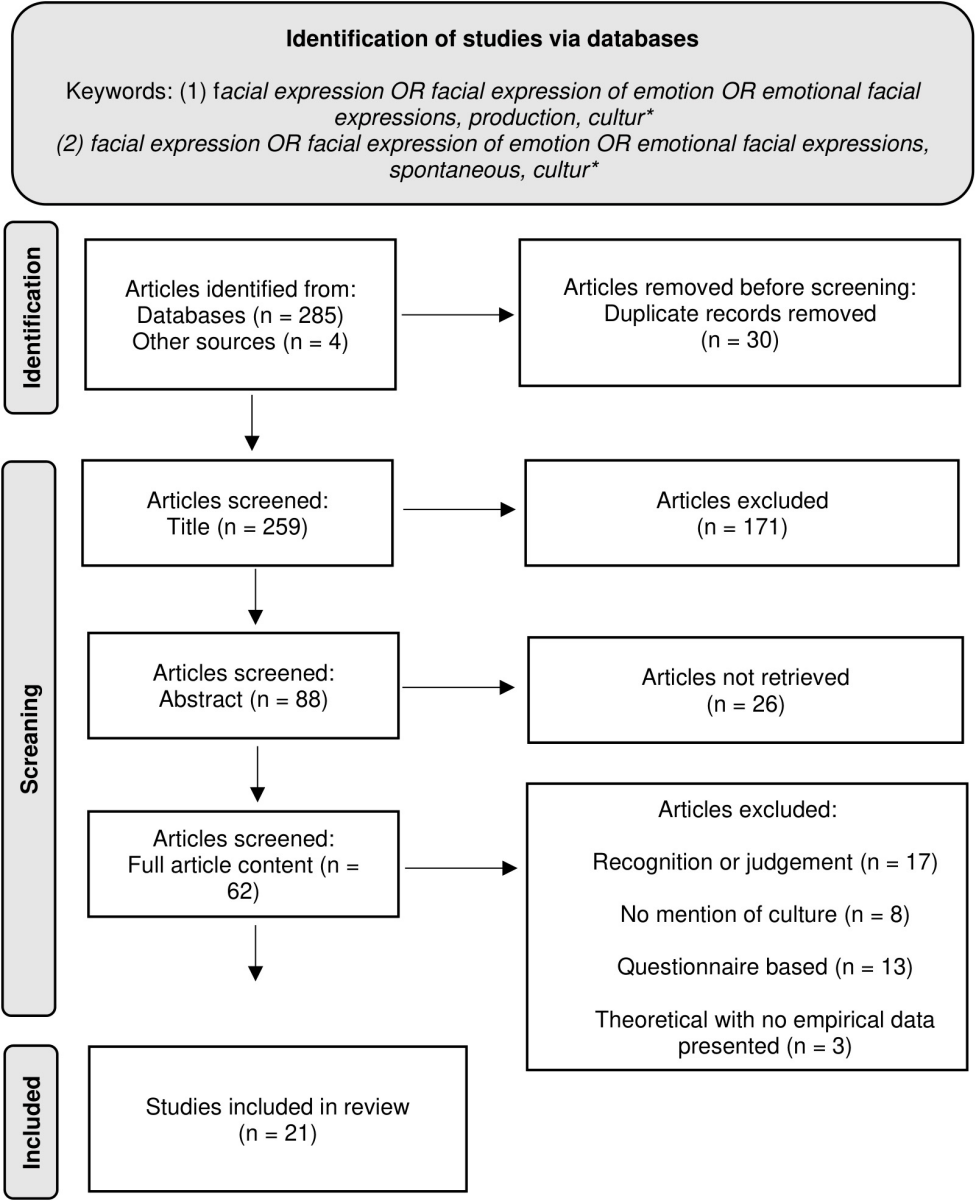
Method diagram. *The term “cultur” was intentionally used in the keywords to capture multiple extensions of the word (e.g., “culture,” “cultural”).

This study was conducted in accordance with the preferred guidelines for systematic reviews and meta-analyses (PRISMA Group; Moher et al., 2009). A systematic review of academic articles published between 1972 and 2024 was conducted through May 10th to May 13th, 2024, drawing upon three databases: PsycINFO, PubMed, and Scopus. The flow diagram illustrating the search strategy employed and screening information is presented in Figure 1. Two search strategies were employed for this review. First, the keywords *facial expressions* OR *facial expressions of emotions* OR *emotional facial expressions, production*, and cultur* (only this prefix was used to include both variations of the word, i.e., culture and cultural) were used. A total of 21 results were obtained with PsycINFO, 126 results with PubMed and 59 results with Scopus. Additionally, a second search was completed combining the keywords *facial expressions* OR *facial expressions of emotions* OR emotional facial expressions, spontaneous* and cultur*. In this second search, the keyword *spontaneous* was employed to access additional articles which touched based on the cultural differences and similarities in the case of emotional facial expressions that are not produced on command or simulated. A total of 19 results from PsycINFO, 30 results from PubMed, and 30 results from Scopus were included. In addition, four additional articles were found through Google Scholar. Before beginning the first screening, duplicates were eliminated, and articles were removed if their title was irrelevant (e.g., not linked to facial expressions). Thus, 30 duplicates were removed, and 171 articles were excluded based on their title.

Based solely on the abstracts, a first screening was conducted to identify irrelevant and relevant articles. Empirical and theoretical articles published in peer-reviewed journals relating to the production of emotional facial expressions in different cultures or ethnicity were included; any articles concerning the influence of other variables exclusively (e.g., age, sex, medical conditions, disabilities), measuring recognition or judgment of emotional facial expressions, and any article not related to facial expressions of emotions were excluded. Finally, animal-related research articles were removed as we are focusing on human behavior exclusively. Based on this first screening, 62 articles were retained. A second screening was conducted in a meeting with the three researchers. Articles were accepted for the review if they were directly related to the production of emotional facial expressions amongst people of different cultures; articles not touching base on such comparison were excluded. The current review thus includes 21 articles, listed in Table 1.

**TABLE 1:** List of selected articles and demographic information.

References	Title	Cultural groups	Sample information (size, age, gender)
Friesen (1972) *	Cultural differences in facial expressions in a social situation: an experimental test of the concept of display rules	Japanese and American	50 undergraduate students (gender unspecified).
Camras et al. (1992)	Japanese and American infants’ responses to arm restraint	Japanese and American	26 5-month-old infants (12 girls); 20 12-month-old infants (8 girls)
Camras et al. (1998)	Production of emotional facial expressions in European American, Japanese, and Chinese infants	Japanese, Chinese, and American	68 infants (38 girls)
Tsai et al. (2002)	Emotional expression and physiology in European Americans and Hmong Americans	Hmong Americans and European American	98 undergraduate students (49 women) 50 Hmong Americans and 48 European American undergraduate students.
Camras et al. (2003) *	Emotional facial expressions in European-American, Japanese, and Chinese infants	European American, Japanese, and Chinese	72 11-month-old infants (36 girls).
Camras et al. (2006)	Culture, ethnicity, and children’s facial expressions: a study of European American, mainland Chinese, Chinese American, and adopted Chinese girls	European American (non-adopted), Chinese (adopted, in USA), Chinese American (non-adopted), Chinese (non-adopted, in China)	163 3-year-olds (all girls)
Matsumoto and Willingham (2006)	The thrill of victory and the agony of defeat: spontaneous expressions of medal winners of the 2004 Athens Olympic Games	North America–Western Europe (Australia, Austria, Belgium, Canada, Spain, France, Great Britain, Germany, Greece, Israel, Italy, The Netherlands, the United States), East Asia (China, Japan, South Korea, Mongolia, North Korea), and all others (Algeria, Argentina, Azerbaijan, Belarus, Brazil, Bulgaria, Cuba, Estonia, Georgia, Iran, Moldova, Poland, Romania, Russia, Slovenia, Tunisia, Ukraine).	84 gold, silver, bronze, and fifth place winners (adults, gender unspecified)
Chentsova-Dutton and Tsai (2007)	Gender differences in emotional response among European Americans and Hmong Americans	Hmong Americans and European Americans	98 college students (49 women)
Elfenbein et al. (2007)	Toward a dialect theory: cultural differences in the expression and recognition of posed facial expressions	Quebecers (Canada) and Gabonese	30 university students (15 women)
Camras et al. (2007)	Do infants show distinct negative facial expressions for fear and anger? Emotional expression in 11-month-old European American, Chinese, and Japanese infants	European American, Japanese, and Chinese	72 11-month-olds (36 girls)
Tracy and Matsumoto (2008)	The spontaneous expression of pride and shame: evidence for biologically innate non-verbal displays	Sighted athletes: North America–Western Europe (Australia, Austria, Belgium, Canada, Spain, France, Great Britain, Germany, Greece, Israel, Italy, The Netherlands, the United States), East Asia (China, Japan, South Korea, Mongolia, North Korea), and all others (Algeria, Argentina, Azerbaijan, Belarus, Brazil, Bulgaria, Cuba, Estonia, Georgia, Iran, Moldova, Poland, Romania, Russia, Slovenia, Tunisia, Ukraine). Blind athletes: 20 countries (unspecified)	140 athletes (adults, gender unspecified)
Matsumoto et al. (2009b)	Sequential dynamics of culturally moderated facial expressions of emotion	North America–Western Europe (Australia, Austria, Belgium, Canada, Spain, France, Great Britain, Germany, Greece, Israel, Italy, The Netherlands, the United States), East Asia (China, Japan, South Korea, Mongolia, North Korea), and all others (Algeria, Argentina, Azerbaijan, Belarus, Brazil, Bulgaria, Cuba, Estonia, Georgia, Iran, Moldova, Poland, Romania, Russia, Slovenia, Tunisia, Ukraine).	84 gold, silver, bronze, and fifth place winners (adults, gender unspecified)
Lewis et al. (2010)	Cultural differences in emotional responses to success and failure	European Americans, African Americans, and Japanese	149 children under 4 years old (82 girls)
Du and Martinez (2015)	Compound facial expressions of emotion: from basic research to clinical applications	Not specified	Pool of images from various sources using the keywords: happy, happily disgusted, happy, disgust, etc., (gender, age, number of participants unspecified).
McDuff et al. (2017)	Large-scale observational evidence of cross-cultural differences in facial behavior	Collectivist and individualist (12 countries, countries not specified)	740,984 adult participants aged 17–65 years old (61% women)
Mui et al. (2017)	Children’s non-verbal displays of winning and losing: effects of social and cultural contexts on smiles	Chinese and Dutch	81 8-year-olds (81 boys).
Cordaro et al. (2018)	Universals and cultural variations in 22 emotional expressions across five cultures	Chinese, Indian, South Korean, Japanese, and American	108 university students (Gender not specified)
Grossard et al. (2018)	Children facial expression production: influence of age, gender, emotion subtype, elicitation condition and culture	Parisian and French Riviera (France)	157 children between 6 and 11 years old (75 girls)
Srinivasan and Martinez (2018)	Cross-cultural and cultural-specific production and perception of facial expressions of emotion in the wild	China, Taiwan, Singapore, United States, Canada, Australia, Great Britain, Iran, Russia, Spain, Mexico, Argentina, Chile, Peru, Venezuela, Colombia, Ecuador, Guatemala, Cuba, El Salvador, Bolivia, Honduras, Dominican Republic, Paraguay, Uruguay, Nicaragua, Costa Rica, Puerto Rico, Panama, Equatorial Guinea.	Pool of images and videos (7.2 million) from web engines (gender, age unspecified)
Sato et al. (2019)	Facial expressions of basic emotions in Japanese laypeople	Japanese	65 adults (44 women)
Fang et al. (2022)	Culture shapes the distinctiveness of posed and spontaneous facial expressions of anger and disgust	Chinese and Dutch	164 university students (106 women)

*Identifies which studies have been retrieved from theoretical article.

Based on the articles selected, we highlight two distinctions in the types of production: spontaneous expressions and posed expressions. The selected articles were categorized based on the type of production used, which is displayed in Table 2. Further information and definitions on these categories will be discussed in greater detail in the following section.

**TABLE 2:** Production type.

References	Production type			
	Spontaneous	Posed	Both (spontaneous and posed)	Other
Friesen (1972)	✓			
Camras et al. (1992)	✓			
Camras et al. (1998)	✓			
Tsai et al. (2002)	✓			
Camras et al. (2003)	✓			
Camras et al. (2006)	✓			
Matsumoto and Willingham (2006)	✓			
Chentsova-Dutton and Tsai (2007)	✓			
Elfenbein et al. (2007)		✓		
Camras et al. (2007)	✓			
Tracy and Matsumoto (2008)	✓			
Matsumoto et al. (2009b)	✓			
Lewis et al. (2010)	✓			
Du and Martinez (2015)				✓
McDuff et al. (2017)	✓			
Mui et al. (2017)	✓			
Cordaro et al. (2018)		✓		
Grossard et al. (2018)		✓		
Srinivasan and Martinez (2018)				✓
Sato et al. (2019)	✓			
Fang et al. (2022)		✓		

## 3 Results

The studies accepted for review are listed in Table 1, with an overview of their sociodemographic information. Based on the articles and their results, four main categories were created regarding production of expressions: spontaneous, posed, both spontaneous and posed, and other. In all categories, studies featuring children/infants and adults are presented to get a broad overview of the effect of cultures on these populations. Within the spontaneous category, we further distinguish between non-naturalistic (laboratory-based) and naturalistic (field or observational) studies. This subclassification highlights contextual differences that allows examination of how culture might modulate emotional facial behavior contexts beyond just a laboratory setting. We added an “other” category since certain production methods did not fit in our previously two defined categories. For instance, some studies are described as “in the wild” where they utilized computer-coding methods to analyze emotional content available on the Internet. Details of the studies are presented in Table 3.

**TABLE 3:** Summary of the main results of each study included in the review.

References	Emotions measured	Production type	Elicitation technique	Coding procedure	Cultural differences found?	If yes; which one?
Friesen (1972)	Masking of negative emotions	Spontaneous	Viewing of stress-eliciting videos.	Facial Affect Scoring Technique (FAST; Ekman et al., 1971)	Yes	While similar array of facial expressions was found, the frequency of positive and negative facial affects differed when an individual was present in the room. When a high-status experimenter was present, Japanese subjects masked their feelings by smiling, while Americans maintained the same facial expressions.
Camras et al. (1992)	Negative affect: distress-pain, anger, sadness, fear	Spontaneous	Arm restraint procedure until facial restraint was met.	Baby Facial Action Coding System (BabyFACS; Oster and Rosenstein, 1996) and the Facial Action Coding System (FACS; Ekman and Friesen, 1978)	No	No cultural differences in the types or distribution of facial expressions, with exception of latency to produce a response.
Camras et al. (1998)	Fear and anger	Spontaneous	Arm restraint procedure and the growling gorilla presentation.	Baby Facial Action Coding System (BabyFACS; Oster and Rosenstein, 1996)	Yes	Differences were observed in the frequency or intensity of certain facial muscles. More Duchenne smiles in American infants, followed by Japanese, and Chinese infants respectively; more intense cry mouths in American infants compared to Chinese; more brow raise in Japanese infants than American; more brow lowerer in American infants than Japanese and Chinese infants.
Tsai et al. (2002)	Happiness, love, pride, anger, disgust, and sadness	Spontaneous	Relived emotion task (Levenson et al., 1991), asked to recall and describe a time where they felt a particular emotion.	Facial Action Coding System (FACS; Ekman and Friesen, 1978)	No	No cultural difference between the two cultures except for the frequency of non-Duchenne smiles: during happiness, fewer Hmong Americans than European-Americans showed this smile.
Camras et al. (2003)	Fear and anger	Spontaneous	Arm restraint procedure and the growling gorilla presentation.	Baby Facial Action Coding System (BabyFACS; Oster and Rosenstein, 1996)	No	No clear relationship between emotions and specific facial configurations, at least within the first year of life, while culture seem to impact intensity.
Camras et al. (2006)	Disgust and negative emotions	Spontaneous	Slide presentation of pleasant, negative images, vinegar presentation (for odor elicitation).	Facial Action Coding System (FACS; Ekman and Friesen, 1978)	Yes	European Americans smiled more than Chinese and American Chinese girls. Adopted Chinese girls produced more disgust expressions than Chinese girls.
Matsumoto and Willingham (2006)	Happiness, contempt, disgust, fear, sadness	Spontaneous	Naturalistic, during the match where they reacted after winning or losing their match.	Facial Action Coding System (FACS; Ekman and Friesen, 1978)	No	Facial expressions expressing victory and defeat evoked by the athletes were similar across all cultures.
Chentsova-Dutton and Tsai (2007)	Anger, disgust, sadness, happiness, pride, and love	Spontaneous	Recall a time where they felt the target emotion very strongly.	Facial Action Coding System (FACS; Ekman and Friesen, 1978)	No	Pattern of differences amongst women and men were similar in both cultural groups.
Elfenbein et al. (2007)	Serenity, contempt, sadness, happiness, shame, anger, disgust, and fear, embarrassment	Posed	Pose their facial expression of emotion following the presentation of a scenario.	Facial Action Coding System (FACS; Ekman and Friesen, 1978)	Yes	Presence of many differences in facial activation were observed for some emotions.
Camras et al. (2007)	Fear and anger	Spontaneous	Arm restraint procedure and the growling gorilla presentation.	Baby Facial Action Coding System (BabyFACS; Oster and Rosenstein, 1996)	No	Largely similar across cultures for both procedures.
Tracy and Matsumoto (2008)	Pride and shame	Spontaneous	Naturalistic, during the match where they reacted after winning or losing their match.	Facial Action Coding System (FACS; Ekman and Friesen, 1978)	Yes	The expression of pride was constantly produced across cultures, while variations were observed for shame behaviors: expressions were less pronounced and intense for participants in individualistic countries who value self-expression (e.g., North America, Europe) compared to individuals in collectivist countries (e.g., China, Japan).
Matsumoto et al. (2009b)	Masking and expression of anger, contempt, disgust, fear, sadness, happiness, and blends of other emotions.	Spontaneous	Naturalistic, during the match where they reacted after winning or losing their match.	Facial Action Coding System (FACS; Ekman and Friesen, 1978)	Yes	Athletes from individualistic cultures expressed their emotions more and athletes from collectivist cultures masked them more. These culturally influenced expressions occurred within a few seconds after initial universal emotional displays.
Lewis et al. (2010)	Shame, embarrassment, pride, sadness.	Spontaneous	During specific games: matching-colored stickers to animal pictures following a key (see Lewis and Ramsay, 2002).	MAX facial movements (Izard, 1995)	Yes	Japanese children expressed less shame, pride, and sadness than European Americans and African American children.
Du and Martinez (2015)	Happily surprised, happily disgusted, sadly fearful, sadly angry, sadly surprised, sadly disgusted, fearfully angry, fearfully surprised, fearfully disgusted, angrily surprised, angrily disgusted, disgustedly surprised, appalled: disgusted angry, hatred: angry disgusted, awed: surprised fearful.	Other	Internet based; coding of images produced spontaneously in various settings.	Facial Action Coding System (FACS; Ekman and Friesen, 1978)	No	17 compound expressions are consistently produced across cultures.
McDuff et al. (2017)	Unspecified	Spontaneous	Viewing of video commercials (e.g., Mars, Coca-Cola, Pepsi).	Facial Action Coding System (FACS; Ekman and Friesen, 2003)	Yes	Participants from individualistic countries displayed more brow furrowing overall, while variations in intensity were observed in different settings.
Mui et al. (2017)	Happiness (Duchenne and non-Duchenne smiles)	Spontaneous	Naturalistic, during a game on a computer (see Shahid et al., 2008).	Facial Action Coding System (FACS; Ekman and Friesen, 2003)	Yes	An interaction was observed in terms of smile intensity and context. Smiles (Duchenne and non-Duchenne) by Chinese children who were teamed up were more intense than those by Chinese children who played alone. The sociality of smile was not observed for Dutch children.
Cordaro et al. (2018)	Amusement, anger, boredom, confusion, contempt, contentment, coyness, desire, disgust, embarrassment, fear, happiness, interest, pain, pride, relief, sadness, shame, surprise, sympathy, triumph.	Posed	Pose their facial expression of emotion following the presentation of a scenario.	Facial Action Coding System (FACS; Ekman and Friesen, 2003)	Yes	Distinct cultural accents were observed for emotions that are more social in nature. 104 unique cultural accents were observed for positive emotions (e.g., open-mouthed lip puckers in India) and negative emotions (e.g., lowering of the head in China).
Grossard et al. (2018)	Happiness, sadness, anger, neutral.	Posed	Producing the expression on request, and imitation of an avatar.	Evaluation of quality according to the ability to be correctly recognized (by independent judges)	Yes	Riviera children performed better than the Parisian children (on a 10-point imitation scale).
Srinivasan and Martinez (2018)	Anger, sadness, surprise, happiness, disgust, and other emotional labels (e.g., outrage, love, loyalty).	Other	Internet based; coding of images and videos produced spontaneously in various settings.	Facial Action Coding System (FACS; Ekman and Friesen, 2003)	Yes	Eight cultural-specific expressions were identified corresponding to 0.05% of possible configurations were found.
Sato et al. (2019)	Anger, disgust, fear, happiness, sadness, and surprise.	Posed	Imitate the facial expression on the photograph and display their expression following the presentation of a scenario.	FaceReader in terms of intensities and action units.	Yes	Intensity and action units expressed varied according to the method of elicitation used. Japanese participants had the potential of producing similar facial movements as Western participants, but they produced expressions more evidently when asked to imitate a photograph then when following a scenario. Variations were also found in the facial activations used, notably for the prototype of disgust, in which the participants did not produce the expression conform with its prototype (e.g., AU9).
Fang et al. (2022)	Anger and disgust	Both	Presentation of the emotion and its scenario (posed) and recall an event that involved disgust or anger (spontaneous).	Facial Action Coding System (FACS; Ekman and Friesen, 2003)	Yes	Expressions were more distinctly produced by Dutch compared to Chinese participants. Three frequent AUs shared between Chinese and Dutch participants’ angry. Posed disgusted expressions of Chinese and Dutch participants shared five frequent AUs. When examining highly frequent action units, results reveal there were fewer marked variations in additional AUs used compared to the results of for the posed expressions.

Among the selected studies, it is worth distinguishing between laboratory-based, naturalistic, and “in the wild” settings. While “in the wild” technically falls under naturalistic observation, these studies relied on pre-existing images (e.g., candid celebrity photos or expressions from photoshoots) without clear information on the elicitation method. Given the ambiguity in these sources, they were grouped separately because it is impossible to clearly know if these expressions are posed or spontaneous. Overall, 15 studies (71.4%) were conducted in laboratory settings, 4 (19.1%) in naturalistic environments, and 2 (9.5%) were classified as “in the wild.” Our results were categorized and interpreted based on the type of elicitation (spontaneous vs. posed) rather than the experimental setting itself, as observed differences were consistently tied to elicitation style. Similar trends were found across both laboratory and naturalistic studies. For instance, masking and intensity effects in the work of Matsumoto (naturalistic) and Camras (laboratory) showed similar patterns, suggesting that context alone (lab vs. real life) does not account for the variability. Laboratory studies of spontaneous expressions typically involve emotionally evocative stimuli that elicit genuine emotional responses, much like those encountered in everyday life. This reinforces the notion that the key distinction lies in whether expressions are posed or spontaneous, rather than in the experimental setting itself.

### 3.1 Spontaneous expressions

A total of 15 studies (71.4%) examined the production of spontaneous expressions, with 7 being with children (46.7%) and 8 with adults (53.3%). In regard to cultures examined, many studies used the eastern and western country classification to regroup countries represented in their study. For example, some studies use Japan and United-States exclusively, while others used a combination of countries in subgroups (i.e., East Asia, Western Europe). See Table 3, column 3 for all the variations of expression production types. Spontaneous expressions generally displayed a high degree of cross-cultural consistency, particularly for emotions such as happiness, sadness, and fear. However, the intensity and frequency of expressions varied across cultural contexts. Studies that elicited production of facial expression through recall of an emotional experience and through presenting various stimuli found this pattern (Chentsova-Dutton and Tsai, 2007; Friesen, 1972; Camras et al., 2006; McDuff et al., 2017). For example, the study by McDuff et al. (2017) observed patterns of spontaneous emotional facial expressions in 12 different countries and found subtle variations in display rules, such as more frequent presentations of brow furrowing in more individualist cultures. Furthermore, individuals from collectivist cultures, particularly East Asian groups, tended to attenuate or regulate emotional expressions more than those from individualistic cultures, consistent with the concept of display rules. This pattern of results was also found when looking at children and infants (Camras et al., 1992, 1998, 2003, 2007). For instance, Camras et al. (2007) observed the emotional facial expression of anger and fear in 11-month-old European American, Chinese, and Japanese infants and they found no major differences. At the same time, studies examining naturally occurring spontaneous expressions, such as those observed in sports competitions, suggest that culturally driven regulation occurs primarily after an initial universal reaction (Matsumoto and Willingham, 2006; Matsumoto et al., 2009b; Tracy and Matsumoto, 2008). For instance, athletes across cultures exhibited similar expressions immediately after a victory or loss, but cultural norms influenced the subsequent modulation of facial behavior. This supports the idea that while the emotion facial expressions of basic emotions tend to be universal, cultural influences guide secondary adjustments in social contexts.

### 3.2 Posed expressions

A total of 4 studies (19%) examined the production of posed expressions, with 1 being with children and 3 with adults. In terms of cultures examined, the most frequent were Chinese (*n* = 2) and Japanese (*n* = 2), followed by, North America (Canada and the USA, *n* = 1, 25%), Gabonese (*n* = 1), Indian (*n* = 1), South Korean (*n* = 1), French (*n* = 1), Dutch (*n* = 1, 25%). Posed emotional facial expressions are deliberate and artificially generated using imitation, commands, or emotion labels. In contrast to spontaneous emotional facial expressions, they exhibited greater cultural variability. In terms of facial muscle activation, studies utilizing the Facial Action Coding System (FACS) found that while core action units for basic emotions were generally consistent, culturally specific facial movements were still found. For instance, Elfenbein et al. (2007) suggest that emotions mainly elicited in social interactions such as anger, contempt, happiness, sadness, serenity, and shame, show greater cultural variations in contrast to those often elicited by internal experience (disgust, fear). Cordaro et al. (2018) expanded on this by analyzing 22 emotions, confirming cultural consistency in core action units but identifying 104 unique cultural accents. For example, open-mouthed lip puckers were more common in India for positive emotions, while head lowering was more frequent in China for negative emotions. Collectivist cultures tended to express emotions in a more inhibited manner (e.g., gaze aversion, head down) (e.g., Tracy and Matsumoto, 2008). Grossard et al. (2018) explored different factors in the production of posed emotional facial expressions amongst children and found regional variations and variations based on the elicitation method. They found variations in the production of emotional facial expressions as a function of the social environment. In Sato et al.’s (2019) study, even though the authors found that Japanese participants had the potential of producing similar facial movements as Western participants, they produced expressions more evidently when asked to imitate a photograph then when following a scenario. Furthermore, they also found variations in the facial activations used. Thus, emotional facial expressions produced with the use of a scenario were not consistent with the prototypical expressions proposed theoretically.

### 3.3 Both (spontaneous and posed expressions)

Fang et al. (2022) compared the production of spontaneous and posed emotional facial expressions, and their findings revealed that Chinese participants’ posed expressions were less distinct than those of Dutch participants, aligning with prior research on cultural influences in facial expressivity (Cordaro et al., 2018; Elfenbein et al., 2007). While both groups shared certain frequent action units (AUs) for anger and disgust, several other AUs varied between cultures. Results also suggest that posed expressions incorporate culturally specific features, whereas spontaneous expressions tend to be more universal. Interestingly, even if spontaneous expressions displayed fewer cross-cultural differences than posed expressions, there were still subtle variations in display rules and intensity. This observation reinforces previous research that collectivist cultures, such as China, exhibit less distinct emotional expressions than individualistic cultures (Matsumoto et al., 2009b; Tsai et al., 2002; Camras et al., 1998).

### 3.4 Other types of production

Beyond laboratory settings, some studies (*n* = 2, 9.5%) have analyzed emotional facial expressions in naturalistic environments using internet-sourced images and videos. The sample of these studies consisted of adults, and the culture groups are presented in Table 1, column 3. Du and Martinez (2015) examined compound emotions (e.g., happy-surprise) using computer-coded facial expressions from global media, identifying 17 compound emotions that were consistently produced across cultures. Similarly, Srinivasan and Martinez (2018) analyzed images from 30 countries and identified eight culturally distinct emotional facial expressions, though their presence varied across linguistic groups. Some expressions were widespread, while others were unique to specific languages or regions.

## 4 Discussion

The present review set out to clarify a longstanding debate in the literature on emotional facial expressions whether these expressions are biologically universal or shaped by cultural influences. While decades of research have documented both striking consistencies and systematic differences across cultural groups, our synthesis highlights that inconsistencies can be traced to methodological factors, particularly the distinction between posed and spontaneous expressions. Most importantly, we found that the production of expressions, long overshadowed by the focus on recognition, plays a critical role in shaping how emotions are conveyed and subsequently interpreted across cultures. By foregrounding production, our review demonstrates that apparent contradictions in the universality versus culture debate may reflect differences in how expressions are elicited, displayed, and regulated, rather than evidence for one theoretical perspective over the other.

### 4.1 Interpretation of findings

Findings from spontaneous expression studies reinforce the idea that basic emotional facial expressions are largely shared between cultures, consistent with the universality hypothesis (Ekman and Friesen, 1971; Ekman et al., 1987; Hwang and Matsumoto, 2015; Izard, 1994). However, cultural variability in intensity and regulation, especially in regulated environments, suggests that socialization plays a critical role in the control of emotional facial expressions (Matsumoto, 1990; Saarni, 1999). A plausible interpretation would be that individuals from collectivist cultures tend to regulate outward expressions to maintain social harmony, while those from individualistic cultures emphasize self-expression (Markus and Kitayama, 1991; Matsumoto et al., 2008). In contrast, posed expression studies demonstrate greater cultural variation in muscle activation and additional facial movements, supporting the idea that culture influences learned expression styles rather than innate emotional responses (Biehl et al., 1997; Jack et al., 2012; Chen and Jack, 2017). The presence of culturally specific facial gestures in posed expressions aligns with previous research on non-verbal accents and dialect theory, suggesting that social norms guide expression production (Elfenbein and Ambady, 2003; Marsh et al., 2003).

Moreover, spontaneous and posed expressions seem to serve different social functions, which may explain why cultural differences are more pronounced in the latter (Buck and VanLear, 2002; Matsumoto et al., 2009a; Namba et al., 2017). More precisely, spontaneous expressions seem automatic and biologically driven, whereas posed expressions seem more consciously controlled, allowing for greater cultural shaping. This distinction is particularly important when interpreting cross-cultural findings on emotional facial expression production.

### 4.2 Theoretical and methodological considerations

The findings raise important questions regarding research methodologies in the field of emotional facial expression production. Studies using posed expressions may overestimate cultural differences, as participants consciously engage in socially influenced expression patterns. Conversely, spontaneous expressions in naturalistic settings, such as those observed in sports competitions, provide stronger evidence for the universality hypothesis. As the literature did not distinguish between modes of induction, it contributed to the debate between universality and cultural differences. However, when we further analyze the modes of production, both positions are legitimate and supported depending on the context. This duality underscores the significance of our contribution, challenging current methodologies and highlighting the need for more nuanced approaches in cross-cultural research.

Additionally, studies examining emotional facial expressions “in the wild” (Du and Martinez, 2015; Srinivasan and Martinez, 2018) using internet-based images and videos highlight potential limitations in data sources, as such media often mix posed and genuine expressions. Future research should refine methodologies to better distinguish between authentic and socially influenced emotional expressions.

Finally, differences in production procedures, particularly whether expressions are posed or spontaneous, may also have significant implications for research on recognition of emotional facial expressions with regards to culture. The type of stimuli used could directly influences how expressions are perceived and interpreted. Consequently, findings of cultural differences or universality in recognition should be considered in light of the production methods underlying the stimuli. Future research should therefore examine recognition as a function of the stimuli used, systematically comparing posed and spontaneous emotional facial expressions, in order to clarify inconsistencies in the current literature and to better inform theoretical accounts.

## 5 Conclusion

In sum, this review demonstrates that the longstanding debate on the universality versus cultural specificity of emotional facial expressions cannot be resolved without careful attention to how expressions are produced. Evidence from spontaneous expression production studies largely supports universality, whereas findings from posed expression studies highlight cultural shaping through socialization, display rules, and non-verbal accents. Rather than being contradictory, these perspectives appear complementary: universality emerges most clearly when emotions are elicited spontaneously, while cultural differences surface when expressions are consciously constructed. Methodological choices, particularly the reliance on posed versus spontaneous stimuli, therefore play a decisive role in shaping theoretical interpretations. Moving forward, research should systematically disentangle these modes of production and examine their consequences for both production and recognition. Doing so will not only clarify inconsistencies in the current literature but also advance theory by situating universality and cultural variation as interconnected outcomes of the interaction between biological mechanisms and cultural contexts.

## Data Availability

The original contributions presented in this study are included in this article/supplementary material, further inquiries can be directed to the corresponding author.
